# Validity of the process and outcome specific measure in women with abnormal Papanicolaou screening test

**DOI:** 10.1371/journal.pone.0325131

**Published:** 2025-06-03

**Authors:** Irena Ilic, Goran Babic, Sandra Sipetic Grujicic, Ivana Zivanovic Macuzic, Ana Ravic-Nikolic, Milena Ilic, Vesna Milicic

**Affiliations:** 1 Faculty of Medicine, University of Belgrade, Belgrade, Serbia; 2 Department of Gynecology and Obstetrics, Faculty of Medical Sciences, University of Kragujevac, Kragujevac, Serbia; 3 Institute of Epidemiology, Faculty of Medicine, University of Belgrade, Belgrade, Serbia; 4 Department of Anatomy, Faculty of Medical Sciences, University of Kragujevac, Kragujevac, Serbia; 5 Department of Dermatovenerology, Faculty of Medical Sciences, University of Kragujevac, Kragujevac, Serbia; 6 Department of Epidemiology, Faculty of Medical Sciences, University of Kragujevac, Kragujevac, Serbia; Wake Forest University School of Medicine, UNITED STATES OF AMERICA

## Abstract

**Introduction:**

Women receiving abnormal Papanicolaou (Pap) findings often face psychosocial burden. This study aimed to evaluate the validity and reliability of the Process and Outcome Specific Measure (POSM) in women with abnormal Pap screening test in Serbia.

**Methods:**

A cross-sectional study was conducted with 238 consecutive women attending cervical cancer screening. Exploratory factor analysis was used to determine the structure of the POSM, and Cronbach’s alpha coefficient to assess internal consistency.

**Results:**

The factor analysis revealed two factors that explained 47.8% of variance: the first – Worry factor (4 items loaded onto the factor represented questions about worries), and the second – Information-Support factor (3 items loaded onto the factor represented questions about feeling informed well enough and being satisfied with support from others). The Cronbach’s alpha coefficients for the Worry factor and the Information-Support factor were 0.572 and 0.428, respectively.

**Conclusion:**

The Serbian version of the POSM could be a useful tool for assessing worry and information-support in women with abnormal Pap smear results.

## Introduction

According to the GLOBOCAN 2022 estimates for cervical cancer incidence and mortality, Serbia ranks eighth in Europe with the age-standardized incidence rate of 13.4 per 100,000 women (after Romania – 21.7 per 100,000 women, the Russian Federation – 17.6, Latvia – 16.9, Bulgaria – 15.4, Ukraine – 15.2, Moldova – 14.2, and Lithuania – 13.7) and ranks sixth with the age-standardized death rate of 6.3 per 100,000 women (after Romania – 9.3 per 100,000 women, Moldova – 7.2, Ukraine – 7.0, and equally – about 6.5 in the Russian Federation and Lithuania) [1]. Over the last decades, organized screening program with the Papanicolaou (Pap) test led to a significant decrease in incidence and mortality of cervical cancer in developed countries, while Serbia was experiencing unfavorable trends in cervical cancer burden [1–3]. Since the 1970s many developed countries reported a decline in incidence trends for invasive cervical cancer, but still failed to reach the World Health Organization’s cervical cancer elimination goals (i.e., 4 per 100,000) [1,4,5]. Serbia introduced population-based cervical cancer screening in 2013 [2].

Globally, invasive cervical cancer was the fourth most common malignancy in women, with ≈663,000 new cases in 2022 (7.2% of all), and >349,000 deaths (8.1% of all) [1]. Same year in Serbia there were 1060 new cases of cervical cancer reported (5.4% of all; ranking as the 4th most common malignancy in women), with 404 deaths (4.6% of all; ranking at the 6th place for mortality) [6]. The noted disparities in cervical cancer incidence worldwide could be due to different levels of economic progress, diversity in lifestyle and socioeconomic distinctions [7,8]. Still, the inequalities in disease burden that are observed across countries are mostly due to a lack of mass screening for cervical cancer [9–11].

Around the world the highest rates of survival from cervical cancer are observed in high-income America and Europe (50–70%) [12]. If cervical cancer is discovered in an early stage, the disease demonstrates one of the highest treatment success rates [13]. In the United States, the localized disease showed a 5-year survival rate of ≈90% in patients aged between 15 and 44 [14]. Overall, Africa has the lowest (<30%), and North America and Northern Europe have the highest (70%) rates of survival [12]. In Serbia, such survival estimates are unavailable.

Screening program for cervical cancer in Serbia is organized and decentralized [15,16]. Cervical cancer screening involves a gynecological examination, speculum examination, Pap test and colposcopy. Cervical cytology swab (Pap test) is the basic test used for screening [15]. If the screening result comes back positive, the doctor who participates in screening informs the women participating in screening and refers her to further diagnostic procedures. In addition, they are let know that during the diagnostics a tissue sample will be taken and hystopathologically examined, which is what will enable making a definitive diagnosis.

Cervical cancer screening, while having a role in the prevention, can cause notable negative psychological consequences in women who receive an abnormal cervical cytology result [17]. An abnormal Pap test can cause hightened stress [18], fear of cancer [19–21], worries regarding health in general [22], fear of dying [23,24], depressive symptoms [25], concerns about having children [26], and being unsatisfied with support from others [27,28]. Also, dimensions of sexual activity had significantly lower values in women with an abnormal Pap test opposed to general population [27]. Additionally, women frequently perceive the Pap test as not needed or useful, or consider themselves as not being in risk to have cervical cancer [29,30].

The psychosocial burden of cervical cancer screening (including worries about general health and cancer-related worries, about sexual intercourse, adverse effects on relationships, concerns regarding fertility, delayed participation in further cervical cancer screening, etc), as well as anxiety and depression, was recorded in some previous studies that were conducted mainly in high income countries [18–21].

Until now, several questionnaires have been used in studies to address the question of psychological burden due to abnormal Papanicolaou smear results, most of which generic. Intriguingly, up to now there have been just a few specific surveys created to assess the psychological effects of receiving a positive screening cervical smear and longitudinal follow-up while undergoing further diagnostic procedures. One of the measures developed is the Process and Outcome Specific Measure (POSM), created in the TOMBOLA (Trial Of Management of Borderline and Other Low-grade Abnormalities) study, to assess the potential psychosocial consequences in women who received a result of low-grade abnormal cervical cytology [31]. The TOMBOLA study was conducted within the routine cervical screening, as a part of the UK Cervical Screening Programmes.

The POSM is a specific 14-item survey for the estimation of the psychosocial status of women who participate in screening for cervical cancer, and it was created in the circumstances of receiving a positive screening result (i.e., borderline and other low-grade cervical smear abnormalities) [31]. Besides this new survey, previously validated ones were also employed within the TOMBOLA study, including the Hospital Anxiety and Depression Scale (HADS) scale [32], the EuroQoL EQ-5D-3L [33] and The Multi-dimensional Health Locus of Control Scale [34].

Studies of the validation of the POSM survey [31,35] using the exploratory factor analysis extracted 2 factors: Factor 1, made of 4 concern-related items showed good reliability with Cronbach’s alpha = 0.769; and Factor 2, made of 3 items on being satisfied with information, support and changes in how women feel about themselves with weaker reliability (0.482). Data gathered at 4 consecutive time points indicated that this factor structure remained stable over time.

Utilizing an appropriate measure to assess psychosocial burden in women with an abnormal result of cervical cytology can provide a deeper understanding and more accurate estimation of the issue, facilitate precise comparisons between countries, and enable the evaluation of different management approaches in cervical cancer prevention. This study aimed to assess the validity and reliability of the POSM questionnaire in women with abnormal findings of Papanicolaou screening test in Serbia.

## Materials and methods

### Study design, study setting, study population

A cross-sectional study was carried out on a cohort of women aged 20–65 who, due to a positive Pap screening test, were scheduled to undergo further diagnostic procedures by a gynecology specialist at the Gynecology and Obstetrics Clinic of the Clinical Center in Kragujevac (No = 279).

Women were eligible for the study if in 2017 they got an abnormal cervical cytology result (as a routine screening test taken under the national Cervical Screening Program) and resided in the Sumadija district of Serbia (No = 260). Exclusion criteria were previous cervical disease, pregnancy at the time of recruitment, illiteracy or inability to speak Serbian well (No = 19). Upon arrival at the clinic, eligible women were provided a leaflet with information about the study, and recruited to participate, on average 6 weeks after receiving the abnormal Pap test. Only women who provided written, informed, and voluntary consent were included. Recruitment took place between January 1st 2017 and December 31st 2017.

## Study sample

Ultimately, 238 (from 260) women meeting the study inclusion criteria were included. Non-participation or refusal (No = 12) was due to not having time or interest, visual difficulties, and insufficient literacy. Additionally, some respondents did not complete the survey or the surveys were not fully filled out, leading to exclusion (No = 10). As a result, the response rate was 91.5%.

### Study sample calculation

The minimum sample size was calculated to be 154 using the Fleiss formula with continuity correction, assuming a power of 80% and a two-sided significance level of 95%. The calculation was made according to data on the prevalence of psychosocial burden in women with positive Pap screening test results from the study by Sharp et al [4]. The Fleiss formula with continuity correction was selected to provide a conservative estimate of sample size, an approach that reduces the risk of underpowering the study and ensures sufficient precision of analyses. The calculations were performed using Epi Info Version 7.2.0.1, provided by the Centers for Disease Control and Prevention, Atlanta.

### Data collection

After providing consent, the women completed (by paper and pencil) an epidemiological survey (which included socio-demographic questions), and psychosocial questionnaires (the POSM, HADS and EuroQoL EQ-5D-3 L) [31–33]. The surveys were filled out in the outpatient clinic of the Gynecology and Obstetrics Clinic, with a nurse or doctor available to help the study participants if needed. The process of filling out the questionnaire took approximately 10 minutes.

### Measures

There are 14 questions in the POSM questionnaire, and these pertain to awareness of one’s own abnormal Pap result (2 questions); own health (5 questions about concern – regarding health, cancer, next test, sex and future fertility; 1 question about delaying pregnancy; 2 questions on sex life); 3 questions regarding cervical screening (beliefs about cervical screening, future intentions for screening and perceived risk of developing cervical cancer); 1 question on being satisfied with received support [31]. Questions offered 5–7 response options on a Likert scale, ranging from “Strongly Agree” to “Strongly Disagree” for most questions; from “Much for the better” to “Much for the worse” for two questions about the change; and from “Much below average” to “Much above average” for the perceived risk issue. Also, two filter questions related to intention to have offspring (“Are you planning to have a child in the future?”) and sexual activity (“Are you sexually active?”) exist, allowing women to skip questions not relevant to them. The questions concern the time period between receiving the abnormal result and filling out the questionnaire. Due to the number of response options differing between POSM items, scores were standardized. Each question had response categories that resulted in a raw score, with a 1–6 range (1–7 for one item with a central neutral option for response). Each item’s raw score was first multiplied by 100, then divided by that item’s highest possible raw score. The factor scores were yielded by summing the standardized scores for all items of that factor, then dividing by the number of items of that factor. Consequently, each factor had scores out of 100. A score for a factor was obtained if the respondents answered all items that make up that factor.

Two filter questions (about the intention to have children and sexual activity) were excluded from the analyses. Also, two questions (“I intend to continue having regular smears”, and “I believe that having regular smears reduces my risk of getting cervical cancer”) were excluded from further analyses, because they are related to belief and intentions regarding screening and could not be scored in the same way as the other questions and since both loaded onto a new, third factor, but this factor had a very low reliability (0.217). Also, the question not related to psychosocial burden after receiving an abnormal cervical cytology result, was therefore deleted from the items and not considered in the analysis (“What do you feel your chances of getting cervical cancer are compared to other women”) as it did not load onto any factor. Additionally, three items (“Since getting my smear result my sex life has changed”, “In the last month I have been worried about my ability to have children in the future” and “Because of the follow-up for my smear I have decided to delay getting pregnant”) showed significant clustering of responses on single response options, and were thus removed from the pool of POSM items.

This left 7 items, representing “core” questions. The scoring system for the POSM demonstrated that a high score was indicative of greater psychosocial burden. According to the authors of the questionnaire [31], the internal consistency of the POSM questionnaire was 0.73 and was rated as acceptable (measured by Cronbach’s standardized alpha coefficient, using cases without missing data).

The HADS is a self-report tool used to screen for symptoms and assess the severity of depression and anxiety in medical settings, primary care, and the general population [32]. The scale has 14 items, seven of which refer to anxiety and seven to depression, with questions referring to the previous week. In our study, the Serbian version of the HADS demonstrated validity and reliability as an instrument for evaluating the psychological impact on women with abnormal Pap smear results [36].

The EuroQoL EQ-5D-3 L [33] is a generic measure of health-related quality of life. In this analysis, the EQ-5D-3L visual analogue scale (VAS) was used, which consists of a scale (resembling a thermometer) numbered from 0 (marking the worst imaginable state) to 100 (marking the best condition imaginable). On that scale, women marked how good or bad their health status was that day.

None of the questionnaires used in the study were part of routine examination, and did not affect in any way the course or outcome of the diagnostic procedures. All questionnaires underwent linguistic adaptation and validation. The translation and cultural adaptation of the original surveys from English to Serbian was carried out according to the internationally accepted methodology [37]: first, translation from the original questionnaire in English into Serbian (“forward” translation) by two professional bilingual translators was done, and then the Serbian version was translated into the original language (i.e., “backward translation”) by a professional translator. The Serbian version of the questionnaire was subsequently tested in a pilot study where several women from the target population participated, i.e., those who participated in cervical cancer screening. This pilot study revealed that the respondents had no difficulty understanding or completing the questionnaire.

### Statistical analysis

According to the Classical Test Theory, data analysis comprised assessment of the internal consistency/ reliability measured by Cronbach’s α reliability coefficient and Item-Test Score Correlation. A value of α coefficient ≥0.7 was considered acceptable. Item-Test Score Correlation is defined as the correlation of an item with the combination of all other items. For judging whether the items within each factor behaved consistently, a correlation >0.2 was considered acceptable.

Validity of the POSM questionnaire was determined using the factor analysis (Principal Components Analysis). Principal component analysis was applied for factor extraction and then factor rotation using appropriate rotation models with Kaiser normalization (delta = 0). The Kaiser-Meyer-Olkin measure and Bartlett’s test of sphericity were performed to test if sufficient common variance and correlation existed between core questions to carry out principal components analysis. A minimum level of 0.5 was used for the Kaiser-Meyer-Olkin test to indicate sufficient common variance. The Kaiser criterion was used to determine the number of statistically significant factors in the principal component analysis (all factors with Eigenvalues greater than 1.0). Probable factors were extracted with the Kattel scatter plot. The total variance of 50% and above determines the good results.

Also, each item’s contribution to the discriminating ability was ascertained with the corrected item-total correlation coefficient. Values of corrected total correlation coefficients <0.2 were regarded as unacceptable, while values ≥0.4 were considered high. Further on, to assess how well the POSM questionnaire was measuring something distinct to that being measured by other questionnaires, each of the POSM items was correlated with the HADS (HADS anxiety and HADS depression) and the EQ-5D VAS scores. The discriminant validity of the POSM scores was evaluated by the Pearson`s correlation.

Statistical analyses were conducted using SPSS 20.0 (IBM SPSS Statistics, Chicago, IL, USA). A significance level of 95% (p < 0.05) was defined for all tests.

### Ethical consideration

This study was approved by the Ethics Committee of the Faculty of Medical Sciences, University of Kragujevac (Ref. No.: 01–2176) and by the Ethics Committee of the Clinical Center Kragujevac (Ref. No.: 01–2869). Voluntary written informed consent was obtained from each participant and conﬁdentiality was protected.

## Results

### Study participants

This study was conducted with 238 consecutive women attending cervical cancer screening. Most of our respondents were under 50 years of age (Table 1). The majority of women (over 80%) reported living in an urban place. Nearly 40% of the respondents were manual workers, while housewives and clerks each represented approximately 20%. More than half of the respondents had a secondary education. Over 80% of the respondents were married at the time of the survey, while around 13% were divorced/widowed.

**Table 1:** Basic socio-demographic characteristics of the respondents in the study (N = 238).

Variables	Number (%)
***Age (years)***	
- ≤ 30	14 (5.9)
-31-40	67 (28.2)
-41-50	77 (32.4)
-51-60	56 (23.5)
- ≥ 61	24 (10.1)
***Residence***	
-Rural	46 (19.3)
-Urban	192 (80.7)
***Occupation***	
-Housewife	50 (21.0)
-Farmer	4 (1.7)
-Worker	90 (37.8)
-Clerk	58 (24.4)
-Expert	36 (15.1)
***Education level***	
-Incomplete elementary school	5 (2.1)
-Elementary school	37 (15.5)
-High school	144 (60.5)
-College	18 (7.6)
-University	34 (14.3)
***Marital status***	
-Single	13 (5.5)
-Married	195 (81.9)
-Divorced/ Widowed	30 (12.6)

### The POSM questionnaire, exploratory factor analysis

To identify any factors within the POSM questionnaire, an exploratory factor analysis was performed on the core questions using the principal components analysis with Varimax rotation. The factor structure of the POSM questionnaire is presented in Table 2. The value for the Kaiser-Meyer-Olkin measure of sampling adequacy for the core questions was 0.599, and the Bartlett test of sphericity had a highly significant (p < 0.001) value, confirming the adequacy of the choice of factor analysis. The two extracted factors explained 47.8% of the cumulative variance. The first factor is the Worry factor (4 items loaded onto the factor represented questions about worries), and the second factor represented Information-Support factor (3 items loaded onto the factor represented questions about feeling well enough informed and being satisfied with support from other people). Both factors corresponded to the original scale. None of the items loaded onto more than one factor. Also, the factor loadings of all items indicated that the association with the factors is strong.

**Table 2:** Exploratory factor analysis with Varimax rotation method for the Process and Outcome Specific Measure (POSM) in women with abnormal finding of Papanicolaou screening test: matrix of correlation coefficients of variables and factors.

POSM item	Worry factor	Information-Support factor	Communalities
Since getting my smear result I have been worried that my next smear will show changes to the cells.	0.788		0.625
Since getting my smear result I have been worried that I may have cervical cancer.	0.722		0.558
Since getting my smear result I have been worried about my general health.	0.623		0.548
Since getting my smear result I have been worried about having sex.	0.528		0.280
In general I feel well enough informed about what my smear result means.		0.782	0.649
Since getting my smear result I have been satisfied with the support I have had from other people.		0.672	0.460
Since getting my smear result the way I feel about myself has changed.		0.566	0.322
Variance explained (%)	27.0	20.8	
Cumulative variance (%)	27.0	47.8	
Kaiser-Meyer-Olkin Measure	0.599		
Bartlett’s Test of Sphericity	Chi-Square = 136.696; df = 21; p < 0.001		

Factor loadings under 0.4 have been suppressed for clarity.

The level of cumulative variance (47.8%) is lower than the acceptable level (50% and above), indicating that the items are not sufficient to explain the model. This cumulative variance shows that there are chances of more factors showing up than the expected factors, which might be linked to the elimination of a number of the variables because of low variable-factor loadings in this study.

**Fig 1:**
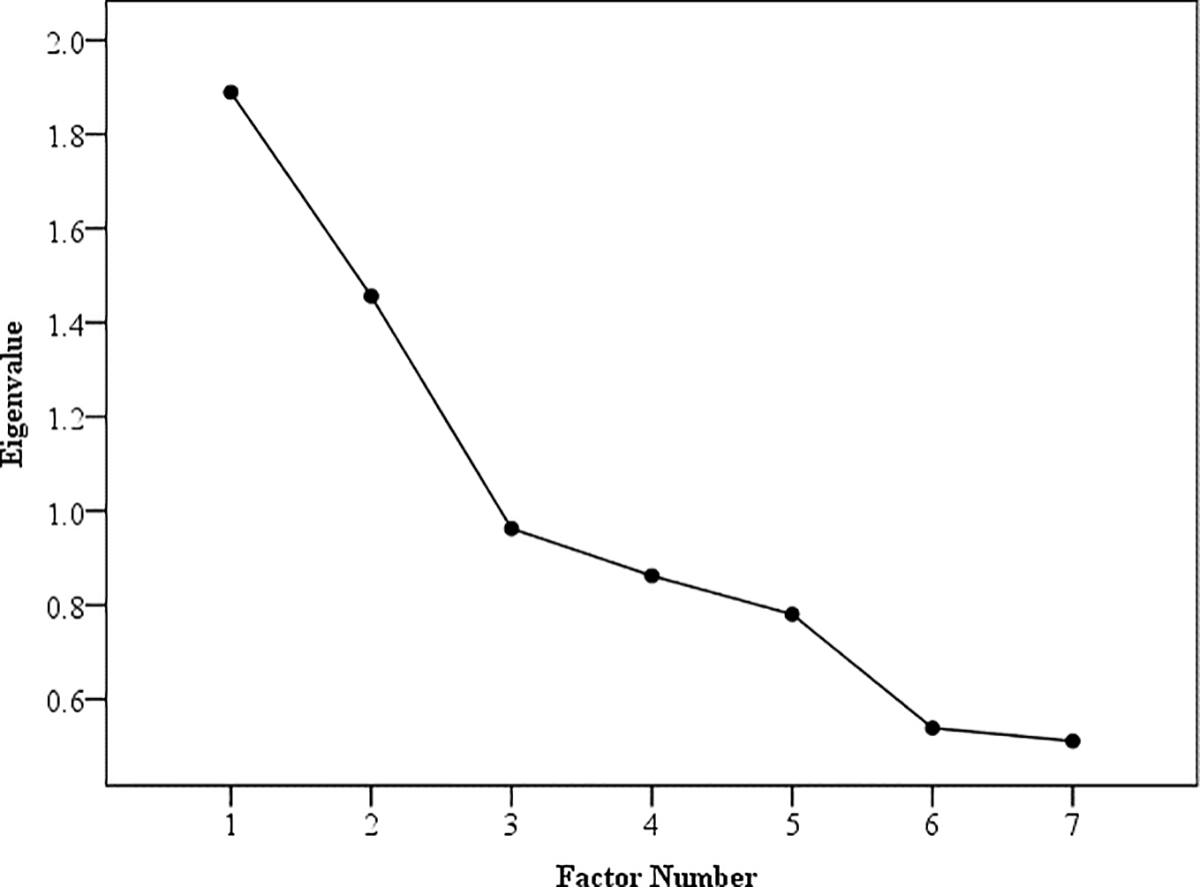
Screeplot of factor in the process and outcome specific measure.

Both Kaiser’s criterion (retention of factors with eigenvalues greater than 1) and Cattell’s Scree plot method (Fig 1) determined extraction of two factors.

### The POSM questionnaire, reliability

The reliability of the Worry factor and Information-Support factor was less favorable (Cronbach’s Alfa Coefficients were 0.572 and 0.428, respectively) (Table 3). Cronbach’s alpha for the overall score for the core questions was 0.464, less than the acceptable level. However, all of the correlations between items and the overall score were acceptable (0.226–441).

**Table 3:** Internal consistency reliability of the Process and Outcome Specific Measure (POSM) in women with abnormal finding of Papanicolaou screening test.

POSM item	Item-Test Score Corelation	Cronbach’s Alfa Coefficient if Item Deleted	Cronbach’s Alfa Coefficient
**Worry factor**			**0.572**
Since getting my smear result I have been worried that my next smear will show changes to the cells.	0.441	0.402	
Since getting my smear result I have been worried that I may have cervical cancer.	0.345	0.467	
Since getting my smear result I have been worried about my general health.	0.333	0.485	
Since getting my smear result I have been worried about having sex.	0.264	0.559	
**Information-Support factor**			**0.428**
In general I feel well enough informed about what my smear result means.	0.309	0.214	
Since getting my smear result I have been satisfied with the support I have had from other people.	0.243	0.360	
Since getting my smear result the way I feel about myself has changed.	0.226	0.387	

### The POSM questionnaire, construct validity

The correlations between the individual items and the Worry factor score were high (in the range between 0.594 and 0.674; p < 0.01) (Table 4). Also, the correlations between individual items and the Information-Support factor score were high (in the range between 0.424 and 0.695; p < 0.01).

**Table 4:** The Process and Outcome Specific Measure (POSM) in women with abnormal finding of Papanicolaou screening test: item-total correlations and correlations with the HADS scales and the EQ-5D VAS.

	Worry factor	Information- Support factor	HADS anxiety	HADS depression	EQ-5D VAS
Since getting my smear result I have been worried that my next smear will show changes to the cells.	**0.656** ^*^	0.038	0.089	−0.057	0.104
Since getting my smear result I have been worried that I may have cervical cancer.	**0.635** ^*^	−0.045	0.059	−0.041	0.133
Since getting my smear result I have been worried about my general health.	**0.594** ^*^	0.107	0.183^*^	−0.008	0.101
Since getting my smear result I have been worried about having sex.	**0.674** ^*^	−0.096	0.186^*^	−0.009	−0.014
In general I feel well enough informed about what my smear result means.	0.007	**0.695** ^*^	−0.048	0.031	−0.004
Since getting my smear result I have been satisfied with the support I have had from other people.	−0.084	**0.424** ^*^	−0.051	0.122	−0.111
Since getting my smear result the way I feel about myself has changed.	−0.096	**0.483** ^*^	−0.013	0.031	−0.090

* Pearson correlation coefficient is significant at the 0.01 level (2-tailed).

Although there were statistically significant correlations between the two POSM items and HADS anxiety sub-scale, the magnitude of the correlation coefficients was relatively low. This result indicates that there is no overlap of the Worry factor/ Information-Support factor with HADS depression.

However, the correlations between the items of POSM and EQ-5D-3L VAS scores were unacceptable. Finally, our study indicated that the POSM measured status different from health-related quality of life and anxiety/depression.

The magnitude of the correlations between the individual POSM questions and the POSM factors was markedly greater than the correlation with the scores of the HADS anxiety and depression subscales and EQ-5D VAS scale (demonstrating good discriminant validity).

## Discussion

The POSM questionnaire was created to evaluate the psychosocial sequelae in women who are undergoing cervical cancer screening procedures. Given the lack of adequate data on the psychosocial burden that women with a positive Pap screening result in Serbia are experiencing, the main aim of this research was to translate the POSM questionnaire from English into Serbian and administer it to women participating in the cervical cancer screening program. In our study, the factor analysis of the POSM questionnaire confirmed the two-factor structure (Worry factor and Information-Support factor) of the original version [31], with a moderate coefficient of internal reliability, slightly below the acceptable level.

To date, quantification of psychosocial burden experienced by women before undergoing additional diagnostic procedures due to a positive cervical cancer screening result has been rare. Namely, some previous studies included the use of qualitative methodology, or had a small sample size, or lacked a specific tool for psychosocial burden assessment, etc. In addition to utilizing an instrument for measuring procedural distress (i.e., psychological distress associated with the medical procedures themselves) in women with a positive cervical cancer screening test (such as the Cervical Dysplasia Distress Questionnaire – CDDQ) [38], utilizing a specific questionnaire that should measure psychosocial burden for both the outcome and procedures is particularly important. In the framework of the TOMBOLA study in the UK [31], a questionnaire was designed aiming to cover the psychosocial sequelae of having received an abnormal cervical smear result and of the subsequent management of the smear. Our study is the first study to assess the validity and reliability of the POSM questionnairein in women before undergoing additional diagnostic procedures due to a positive cervical cancer screening result.

Globally, limited data on the validation of the POSM questionnaire are available in the existing literature. Since the original validation [31], only a few studies have utilized the POSM survey. To date, the POSM has been applied in research involving women with low-grade abnormal cytology results [31,39], vulval intraepithelial neoplasia (VIN) [40] and micro-invasive cancer of the cervix uteri [41].

In this research, the Cronbach’s alpha for the POSM questionnaire was 0.464, comparing unfavourably with the internal consistency reported in the original study (0.73) [31], and also with each of the subscales of HADS in this study (0.862 for the anxiety and 0.851 for the depression subscale) [36]. In women with VIN, Cronbach’s alpha coefficient for the POSM was 0.69 [40]. The reliability measures for the factors of POSM were somewhat lower, which could at least in part be because of the small number of items within both factors. Also, strong statistically significant correlations existed between some of the individual items of the VIN survey and the total score of POSM, demonstrating convergent validity. Thus, even though the POSM was used for women with low-grade abnormal cytology, it is also applicable to issues of concern to women with VIN. Also, in women with vulvar intraepithelial neoplasia, the VIN question regarding support from friends and/or family successfully differentiates from psychosocial burden measured by POSM total score [40]. Differences in psychometric characteristics of the POSM questionnaire between some studies could be attributed to differences in the diseases studied, differences in degree of severities of cervical abnormality, as well as differences in socio-demographic and other characteristics of the studied populations [21], while some questionnaires were utilized as specific to certain management [31], or as uni-dimensional (limiting the focus to a single facet of psychosocial consequences, i.e., psychosexual issues, cancer-related fear, concern about future offspring, worry about infectivity) [42–44].

Even though it was originally created to evaluate the psychosocial burden of getting a low-grade abnormal cervical smear result, the POSM survey has also been employed in population of women with high-grade abnormalities or microinvasive disease to assess the concerns on sexual activity, fertility, and developing cervical cancer in the future [41]. Comparing concerns of women with microinvasive cervical cancer and those with high-grade cervical intraepithelial neoplasia, utlizing the POSM, did not show significant differences in terms of sexual activity, fertility, and developing cervical cancer in the future [41]. Cairns and coauthors [41] did not find significant differences about ongoing concerns during follow-up between women who have microinvasive cancer and those who have high grade cervical intraepithelial neoplasia, a finding that could be because of the small number participants in the study.

Consistent to the original validation study [31], our research confirmed the discriminant validity of the POSM questionnaire: core POSM questions demonstrated stronger correlation with the factors of POSM (Worry factor and Information-Support factor) than with anxiety and depression (as measured by HADS) or health-related quality of life (as measured by the EQ-5D-3L VAS). The findings of this study suggest that the POSM survey provides adequate information about the psychosocial burden faced by women. Still, to fully assess the validity and reliability of the POSM questionnaire, it is necessary to carry out more studies in the future.

### Strengths and limitations of the research

To the best of our knowledge, this is the first validation study of the Serbian version of the POSM questionnaire. Also, it is one of the few validation studies of the POSM survey globally. There are not studies in the available literature worldwide that have examined psychosocial burden in women with a positive Pap screening test undergoing additional diagnostic procedures. The main strength of this study includes its assessment of both reliability and validity. Additionally, the participation rate of our study was satisfactory, at 91.5%. However, there are several potential limitations. First, the results are based on self-reported data. Second, the study employed a cross-sectional design, with all known associated shortcomings. Third, different types of bias (i.e., information bias, non-response bias, response bias) may be present. Our study sample comprised women from one district in Serbia: this study included women through recruitment at the only gynecological clinic for the entire population covered by screening, which indicates that all women had the same chance to participate in the study. Consequently, the sample was not selected, which suggests that the study sample is representative of the entire population covered by cervical cancer screening in Kragujevac. Additionally, the subjects included in this study are representative of the observed population thanks to having this cross-sectional study carried out on a cohort of 238 consecutive women attending cervical cancer screening, the sample size being much larger than the calculated minimum sample size, and the response rate being high. But despite all of that, the issuess of sample representativeness are always present, either due to recruitment barriers or concerns of confidentiality.

## Conclusions

The Serbian version of the POSM questionnaire could serve as a useful tool for assessing the psychosocial burden in women with abnormal Pap smear screening results. This study showed that, in women undergoing cervical screening procedures, the POSM questionnaire successfully differentiates psychosocial burden from health-related quality of life and anxiety/depression. The two factors of POSM questionnaire (Worry factor and Information-Support factor) showed moderate internal reliability. Employing the POSM scale could lead to a deeper understanding and more precise evaluation of the psychosocial impact of cervical screening across different countries. By using an appropriate measurement tool to assess the psychosocial burden in women with abnormal cervical cytology results, it is possible to estimate more accurately the extent of the issue, and evaluate the effects of different management strategies in cervical cancer prevention.
